# Evaluation of the Physical and Mechanical Properties of a New Composite Resin: An In Vitro Study

**DOI:** 10.7759/cureus.114619

**Published:** 2026-08-16

**Authors:** Claudia Melo, Cleire Mejia, Herney Garzón

**Affiliations:** 1 Oral Rehabilitation, Universidad del Valle, Cali, COL

**Keywords:** elastic modulus, light cure composite, nanofiller composite, resin composite, vickers test

## Abstract

Introduction

As a versatile treatment alternative, dental resins require a thorough understanding of their physical and mechanical properties to ensure proper selection and clinical efficacy.

Objective

To evaluate the physical and mechanical properties of a novel dental resin in comparison with commercially available resins.

Methods

This in vitro study evaluated Vickers hardness, elastic modulus, and depth of cure. Five specimens per test were prepared for each of the following commercial brands: Filtek Z250 Universal Restorative (3M), Empress Direct (Ivoclar), and New Stetic resin, yielding a total of 45 specimens. All specimens were fabricated by a single, standardized operator. Testing was performed using an Indenta Met microhardness tester (Model 1104; Buehler Ltd., Lake Bluff, IL), a universal testing machine (Model 3366; Instron Corp., Norwood, MA), and an analog micrometer (Ubermann), respectively. Statistical analysis was conducted using the Kolmogorov-Smirnov test, one-way ANOVA, and Tukey's HSD post-hoc test to contrast the evaluated properties across brands. Statistical significance was set at p < 0.05.

Results

Filtek Z250 Universal Restorative (3M) exhibited the highest Vickers hardness (128.98 VHN) and depth of cure (2.87 mm), followed by New Stetic (110.93 VHN; 2.64 mm) and Empress Direct (Ivoclar; 64.61 VHN; 2.03 mm), demonstrating statistically significant differences in both tests (p < 0.000). Regarding the elastic modulus, New Stetic exhibited the highest stiffness (3,377.50 MPa), Filtek Z250 (2,401.33 MPa), and Empress Direct (2,277.31 MPa). Although overall differences were found among the groups (p < 0.000), no statistically significant difference was observed between Filtek Z250 and Empress Direct (p = 0.743).

Conclusions

The three composite resins evaluated in this study exhibited distinct variations in their physical and mechanical properties. Nevertheless, the performance of the New Stetic resin fell within established standards for clinical quality and normality, demonstrating that this biomaterial represents a viable alternative for restorative treatments.

## Introduction

Dental caries is a multifactorial disease characterized by the destruction of enamel, dentin, and/or cementum, with an etiology generally attributed to acid-producing bacteria within the oral cavity [1]. Historically, dental amalgam has been widely utilized as a restorative material due to its low cost and rapid manipulation. Nevertheless, because amalgam contains mercury and given its irreversible environmental and health effects, the Minamata Convention established a global phase-down and elimination of its use, originally projected for 2023 [2].

Composite resins are defined as highly cross-linked polymeric materials reinforced by a dispersion of amorphous silica, glass, crystalline, or organic resin filler particles, and short fibers bonded to the matrix by a coupling agent [1,3]. These biomaterials are of paramount importance in restorative dentistry, having proven to be ideal due to their excellent aesthetic outcomes and ease of clinical manipulation. Over time, various types have been developed and classified based on filler particle size and distribution: macrofilled resins contained large particles (>1 μm; 70-75% by weight), which caused excessive wear and subsequent clinical failure. Later generations featured smaller filler particles and are currently categorized into three main types: microfilled, microhybrid, and nanohybrid [4]. Notably, the incorporation of nanotechnology, particularly nanoclusters, has enabled the development of composites with enhanced physical and mechanical properties [5].

Given the high demand for these biomaterials and the emergence of novel composite resins, analyzing their physical and mechanical behavior is essential to determine their clinical indications for anterior and posterior restorations [1,5]. These materials must withstand masticatory cycles involving tensile, compressive, and shear forces, as well as environmental factors, such as temperature fluctuations and pH changes [5-8]. Additionally, they are subject to histological factors regarding dental tissue quality [9], caries risk, patient habits, and psychosocial factors [10]. Therefore, evaluating their in vitro performance through specific tests, such as elastic modulus, depth of cure, and Vickers hardness, is necessary [11]. The incorporation of novel components that enhance both their structure and clinical performance has driven their evolution, aiming to minimize common failure causes, such as material fracture, secondary caries, staining, and wear, thereby ensuring excellent clinical performance and treatment longevity [4,6].

This study aimed to evaluate the physical and mechanical properties of a Colombian-manufactured dental resin to compare its quality against other imported dental resins.

This article was previously presented as a meeting abstract at the XXIII Research Symposium of the Faculty of Health, October 19-21, Cali, Colombia; XXXI ACFO National Meeting of Dental Research, November 3-5, 2021, Bogotá, Colombia; ENIO 2021, XXIX National and XX Ibero-American Meeting of Dental Research, November 17-19, 2021; and Virtual Modality, Autonomous University of Yucatan - México.

## Materials and methods

This was an in vitro, observational, experimental study that evaluated three properties. Vickers hardness testing was conducted following ISO 4049 standards at the Laboratory for Materials Research and Basic Sciences (LIMACIB) of the Faculty of Dentistry at the Universidad Nacional de Colombia. Elastic modulus testing was processed at the Biomaterials Laboratory of the Fundación Universitaria UniCIEO, and depth of cure testing was performed at the Faculty of Dentistry of the Universidad del Valle.

The specimens were fabricated by a single standardized operator utilizing a composite placement instrument (FP3 spatula) and the specific molds designated for each evaluation. Fabrication was conducted under controlled environmental conditions at a temperature of 23.0 ± 1.0°C and a relative humidity between 30.0% and 70.0%. For each of the three mechanical tests, a sample size of 5 (33.3%) specimens was included for each brand: Filtek Z250 Universal Restorative (3M Oral Care, St. Paul, MN), Empress Direct (Ivoclar Vivadent, Schaan, Liechtenstein), and the New Stetic experimental resin (New Stetic, Medellín, Colombia), yielding a total sample size of 45 (100.0%) specimens [12-14]. Table 1 presents the evaluated materials.

**Table 1:** Evaluated materials Particle size is expressed in micrometers (μm) and nanometers (nm). Monomers incorporated into the inorganic matrix: Bis-GMA: bisphenol A diglycidyl ether dimethacrylate; TEGDMA: triethylene glycol dimethacrylate; UDMA: urethane dimethacrylate; Bis-EMA: ethoxylated bisphenol A dimethacrylate. Flexural strength is measured in megapascals (MPa), polymerization time is determined in seconds (S), and the increment depth of each resin is measured in millimeters (mm).

Composite resin	Classification	Flexural strength	Chemical composition	Polymerization time	Increment depth
Dental resin New Stetic, Shade A2	Nanohybrid	120 MPa	Bisphenol A diglycidyl ether dimethacrylate (Bis-GMA): 1-10%, triethylene glycol dimethacrylate (TEGDMA): 1-10%, urethane dimethacrylate (UDMA): 1-10%, aluminosilicates, silica, silane-treated inorganic filler: 70-80%, initiators and additives: 1-2%	20 s	< 2.5 mm
Filtek Z250 Universal Restorative 3M/Solventum, Shade A2	Microhybrid (0.6 μm)	150 MPa	Zirconia/silica (75-85% by weight), bisphenol A diglycidyl ether dimethacrylate (Bis-GMA), triethylene glycol dimethacrylate (TEGDMA), urethane dimethacrylate (UDMA), and ethoxylated bisphenol A dimethacrylate (Bis-EMA)	20 s	< 2.5 mm
Empress Direct Ivoclar, Shade A2	Nanohybrid (550 nm)	115 MPa	Barium glass filler, mixed oxides, and barium aluminum fluorosilicate glass: 77.5-79%. Dimethacrylate: 20%. Ytterbium trifluoride: 9.8%. Prepolymer: 19.6%. Highly dispersed silicon dioxide. Catalysts and stabilizers: 0.4%. Pigments: <0.1%	10 s	1.5 mm

Vickers hardness

**Figure 1:**
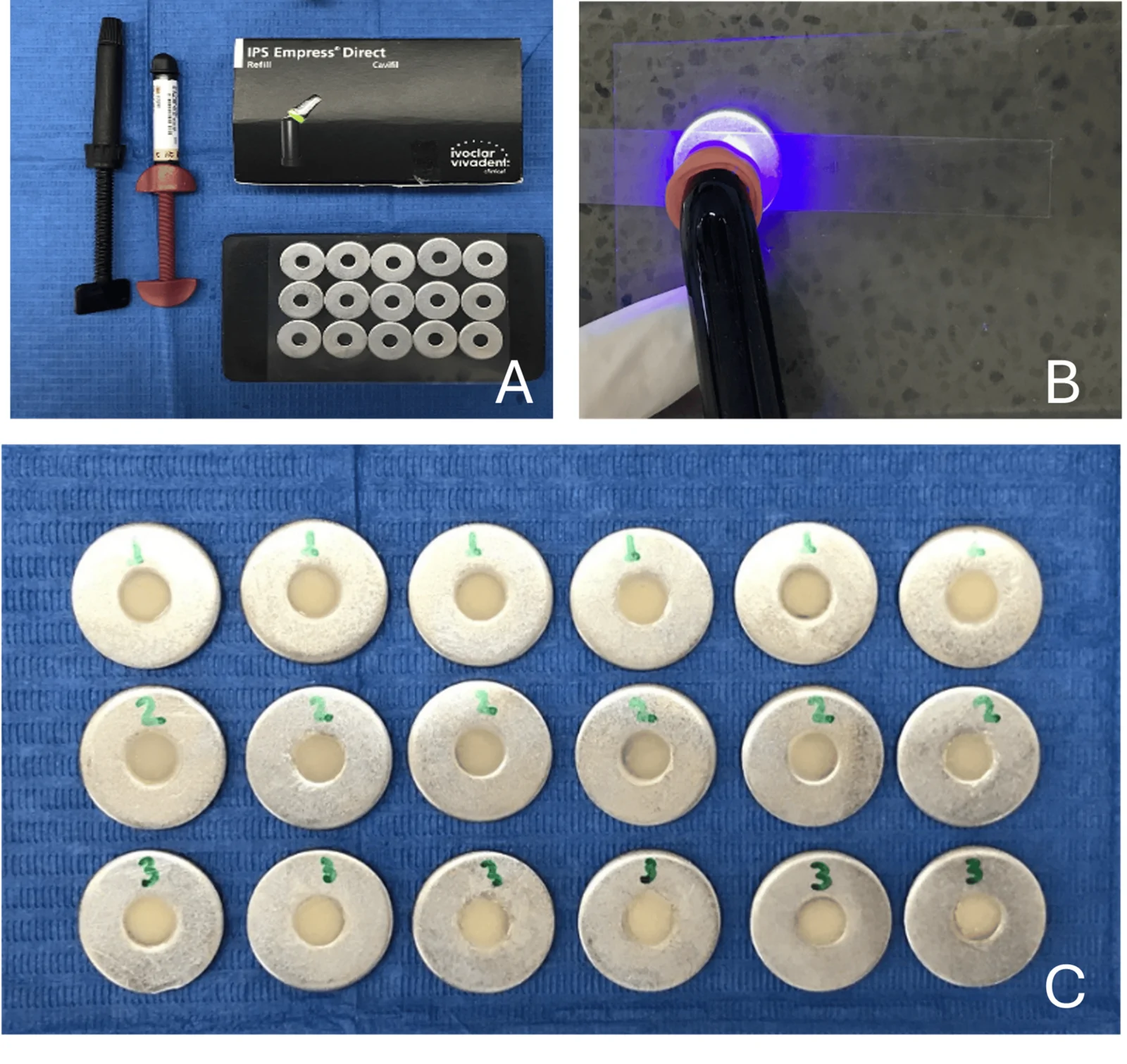
Vickers hardness sample preparation (A) From left to right, the resins are the New Stetic test resin, 3M Filtek Universal Z250, and Ivoclar Empress Direct. (B) Photopolymerization was performed for 20 s using a VALO Grand LED curing light (Ultradent Products, Inc., South Jordan, UT) at an irradiance of 1,000 mW/cm^2^. (C) Fifteen specimens measured 2 mm × 6 mm

Cylindrical specimens measuring 2 mm × 4 mm were fabricated, whereas depth-of-cure specimens measured 2 mm × 6 mm (Figure 1C). Photopolymerization was performed for 20 s using a VALO Grand LED curing light (Ultradent Products, Inc.; South Jordan, UT) at an irradiance of 1,000 mW/cm^2^ f (Figure 1B).

**Figure 2:**
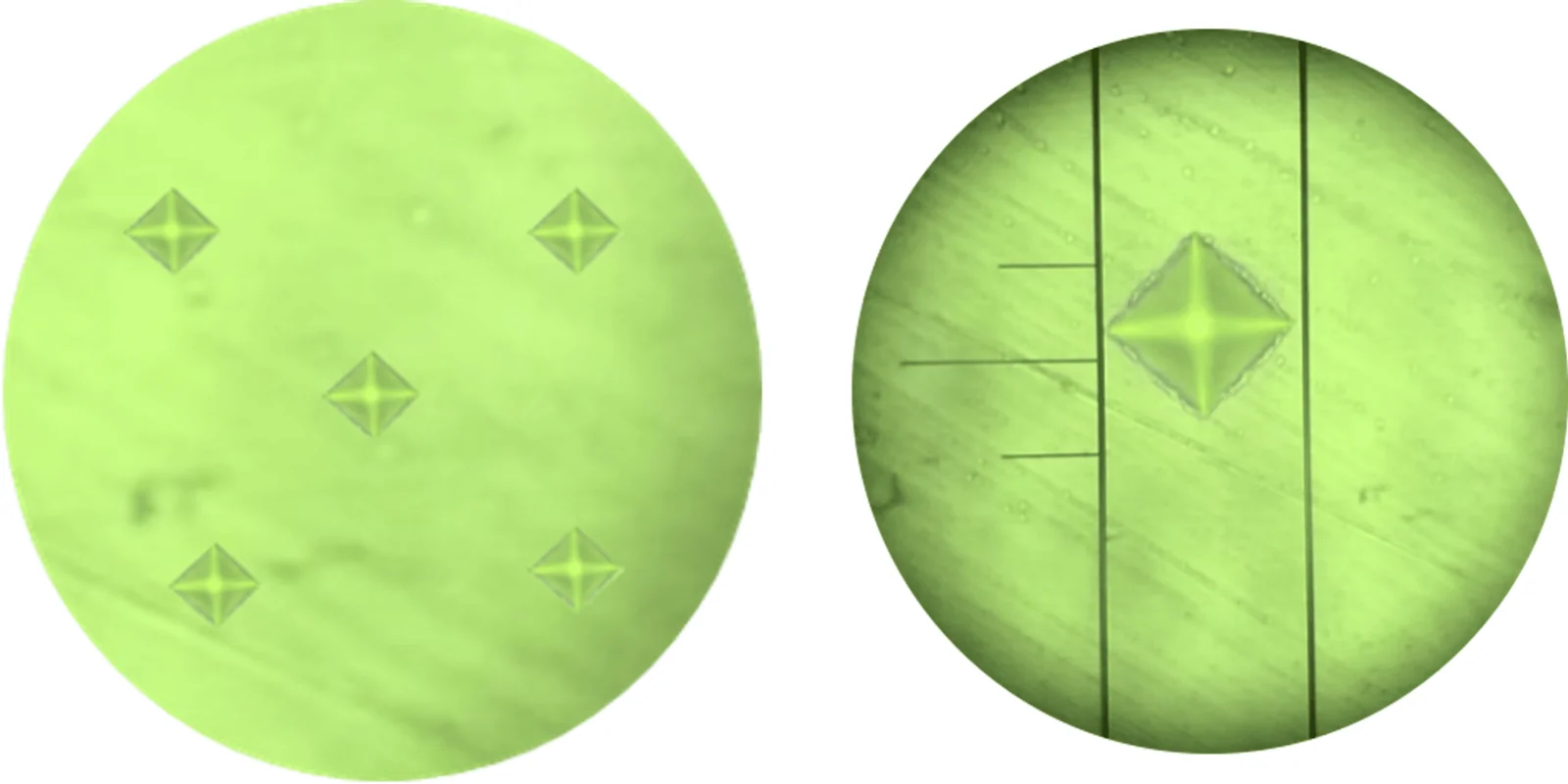
Vickers microhardness indentation analysis

The specimens were stored for 24 h prior to testing. Vickers microhardness was evaluated using an Indenta Met tester (Model 1104; Buehler, Ltd., Lake Bluff, IL), programmed with a 200 g load and a 10 s dwell time. Five indentations were performed per specimen, spaced 1 mm apart. The indentations were subsequently analyzed under an optical microscope at 55× magnification to measure diagonal lengths for data registration and statistical analysis (Figure 2).

Elastic modulus testing 

**Figure 3:**
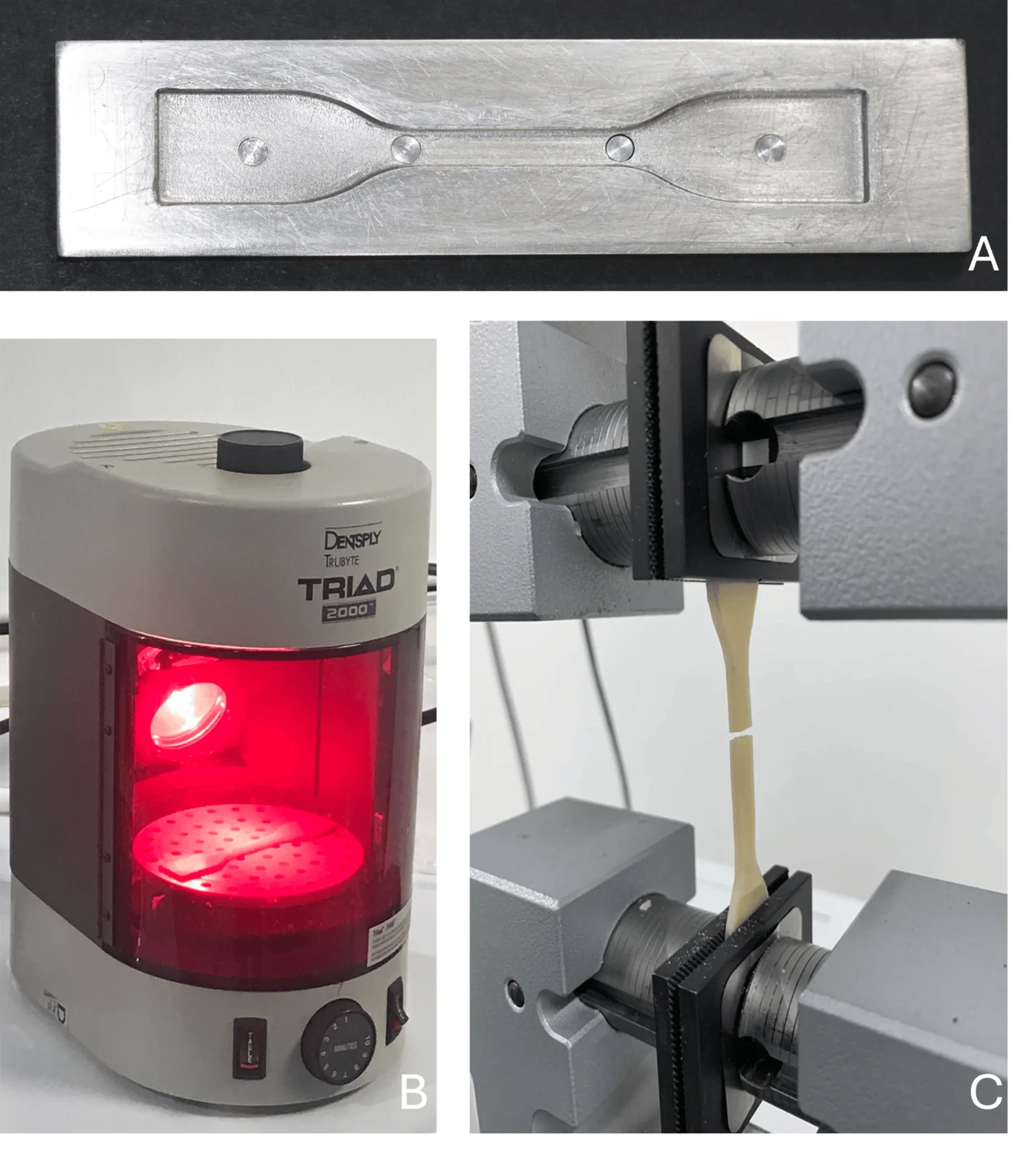
Elastic modulus testing (A) Type IV matrix. (B) Triad II curing oven (Dentsply Sirona, Inc, Charlotte, NC). (C) Universal testing machine (Model 3366; Instron Corp., Norwood, MA)

The specimens were fabricated using a type IV matrix and subjected to an additional one-minute post-curing cycle in a Triad II curing oven (Dentsply Sirona, Inc., Charlotte, NC). The elastic modulus specimens were then subjected to a tensile load using a universal testing machine (Model 3366; Instron Corp., Norwood, MA) configured with a load range of 0-100 kg at a crosshead speed of 1 mm/min (Figure 3).

Depth of cure

**Figure 4:**
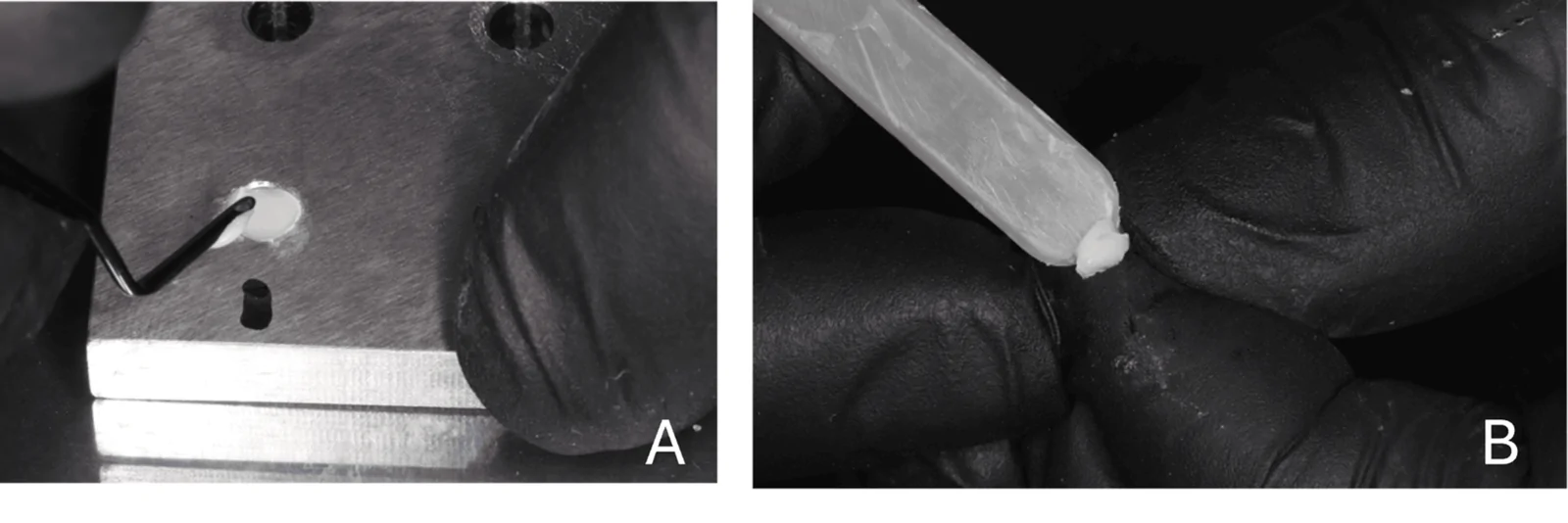
Depth of cure specimens The operator fills the stainless steel mold measuring 6 mm in length and 4 mm in diameter, with each of the resins (A), and once polymerization has occurred, the unpolymerized material is removed (B).

**Figure 5:**
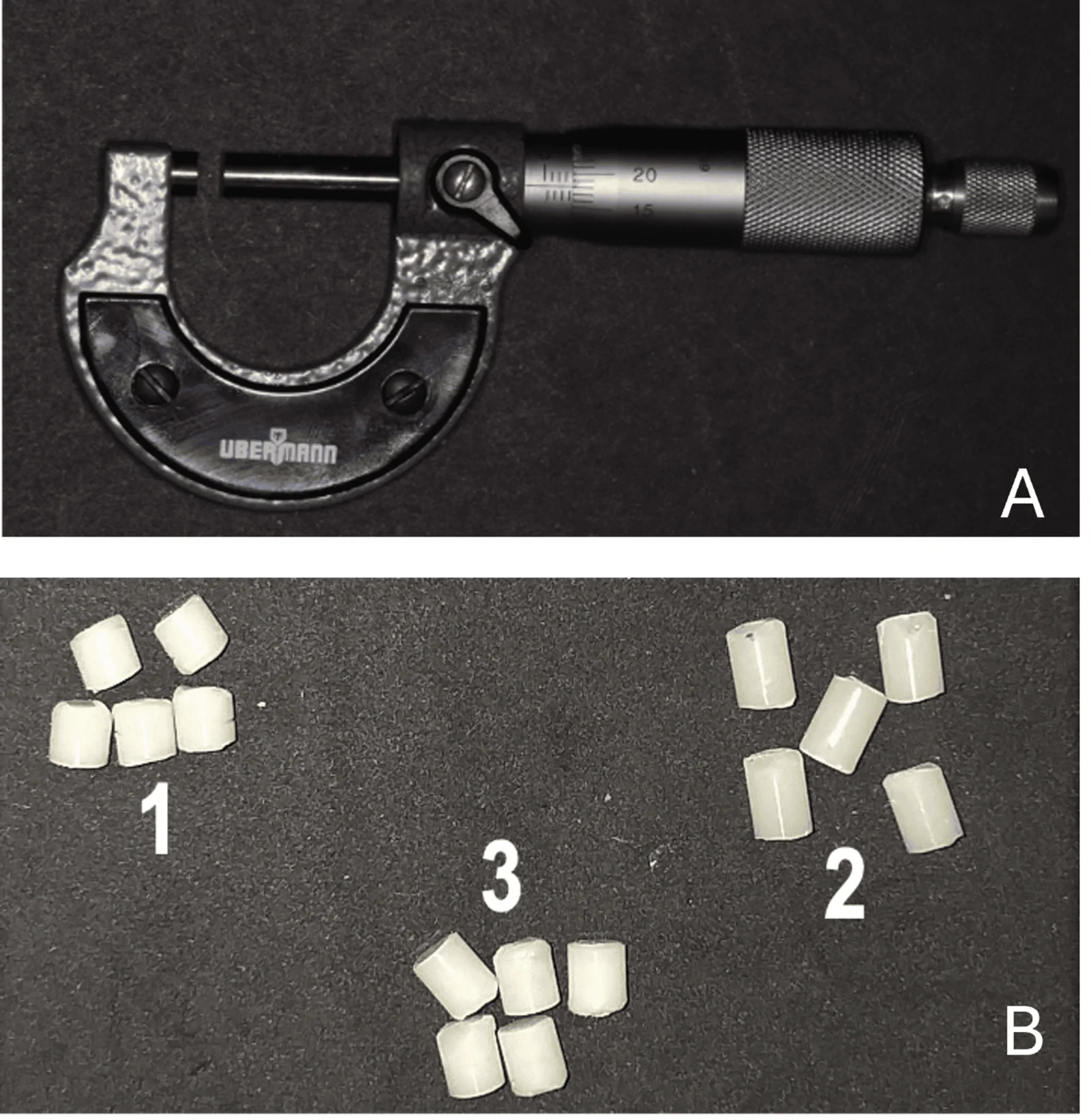
Depth of cure specimens (A) Ubermann analog micrometer calibrated. (B1) Specimens of Empress Direct Ivoclar, shade A2. (B2) Specimens of dental resin New Stetic, shade A2. (B3) Specimens of Filtek Z250 Universal Restorative 3M/Solventum, shade A2.

Specimens were prepared using a stainless steel mold measuring 6 mm in length and 4 mm in diameter (Figure 4). Photopolymerization was performed with a VALO LED curing light (Ultradent Products, Inc.) at an irradiance of 1,000 mW/cm^2^, following the exposure times recommended by the manufacturer. The height of the polymerized composite cylinder was subsequently measured using an Ubermann analog micrometer (Figure 5). 

Statistical analysis

A total of 45 specimens exhibiting homogeneous surfaces, free of fractures and delamination, and within the prescribed dimensions were included. Statistical analysis was conducted using the Kolmogorov-Smirnov test, one-way ANOVA, and Tukey's HSD post-hoc test to evaluate and contrast the properties across brands. All data were interpreted with statistical significance set at p < 0.05.

## Results

Vickers hardness

One-way ANOVA revealed a statistically significant difference in the hardness evaluation metrics among the groups (p < 0.05), demonstrating that the chemical composition and particle configuration significantly influenced the material's resistance to indentation. The Filtek Z250 Universal Restorative group (3M) achieved the highest mean hardness value, 128.99 VHN, among all evaluated materials. When comparing the nanohybrid materials, the New Stetic experimental resin (New Stetic) presented a higher mean hardness value, 110.93 VHN, than the Empress Direct control group (Ivoclar), 64.62 VHN (Table 2). Despite these performance discrepancies, all three composite resins complied with the minimum mechanical quality thresholds established by the reference technical standards.

**Table 2:** Mean values of the mechanical and physical properties of the tested materials Values are presented as mean ± standard deviation (SD) derived from the experimental groups (n = 15 per material). Hardness is expressed as the Vickers hardness number (VHN); elastic modulus is expressed in megapascals (MPa); and depth of cure is expressed in millimeters (mm). Statistical analysis was conducted using the Kolmogorov-Smirnov test, one-way ANOVA, and Tukey's HSD post-hoc test, with the statistical significance threshold set at p < 0.05.

Composite Resin	Depth of Cure (mm), Mean ± SD	Vickers hardness (VHN), Mean ± SD	Elastic modulus testing (MPa), Mean ± SD
Filtek Z250 Universal Restorative 3M/Solventum, Shade A2	2.87 ± 0.046	128.98 ± 10.67	2401.33 ± 421.18
Empress Direct Ivoclar, Shade A2	2.03 ± 0.027	64.61 ± 0.51	2277.31 ± 77.83
Dental resin New Stetic, Shade A2	2.64 ± 0.056	110.93 ± 4.56	3377.51 ± 159.55
p-value (one-way ANOVA)	<0.001	<0.001	0.743

Elastic modulus testing

Upon conducting this evaluation, the composite resins exhibited unequal values among themselves, reflecting a different performance trend from that observed during the surface hardness and depth of cure testing. Regarding this specific test, it was observed that the New Stetic experimental resin (New New Stetic) achieved an elastic modulus value of 3,377.51 MPa, demonstrating greater stiffness in comparison to the commercial control groups, Filtek Z250 Universal Restorative (3M), which recorded 2,401.33 MPa, and Empress Direct (Ivoclar Vivadent), which recorded 2,277.31 MPa. According to the inferential one-way ANOVA statistical test, a statistically significant difference was found in the global results of this evaluation (p < 0.05); however, the pairwise multiple comparisons post-hoc test indicated an absolute absence of a statistically significant difference (p = 0.743) between both commercial control resins, thereby corroborating their mutually equivalent behavior of lower stiffness during this mechanical test (Table 3).

**Table 3:** Tukey's HSD post-hoc test for elastic modulus values Values are presented as mean differences (I-J) in megapascals (MPa), with standard errors and 95% confidence intervals, derived from Tukey's honestly significant difference (HSD) post-hoc multiple comparisons following a statistically significant one-way ANOVA (n = 15 per material). *The mean difference is significant at the 0.05 level.

Composite Resin (I)	Composite Resin (J)	Mean Difference (I-J) (MPa)	Std. Error	p-value	95% CI Lower	95% CI Upper
Empress Direct Ivoclar, Shade A2	Dental resin New Stetic, Shade A2	-1100.20*	166.90	<0.001	-1545.45	-654.94
	Filtek Z250 Universal Restorative 3M/Solventum, Shade A2	-124.02	166.90	0.743	-569.27	321.23
Dental resin New Stetic, Shade A2	Empress Direct Ivoclar, Shade A2	1100.20*	166.90	<0.001	654.94	1545.45
	Filtek Z250 Universal Restorative 3M/Solventum, Shade A2	976.18*	166.90	<0.001	530.92	1421.43
Filtek Z250 Universal Restorative 3M/Solventum, Shade A2	Empress Direct Ivoclar, Shade A2	124.02	166.90	0.743	-321.23	569.27
	Dental resin New Stetic, Shade A2	-976.18*	166.90	<0.001	-1421.43	-530.92

Depth of cure

The depth of cure test allowed us to evaluate whether the experimental testing resin complies with the standard parameters indicated in the technical regulations. According to the obtained values, the resin reflects an adequate behavioral performance by achieving a layer polymerization thickness of up to 2.64 mm, a value that is close to the one achieved by the commercial control resin Filtek Z250 Universal Restorative (3M Oral), which recorded 2.87 mm, and superior to the one achieved by the Empress Direct control resin (Ivoclar Vivadent) (Table 2). These experimental values clearly evidence an optimal conversion of the monomers found within the organic matrices of each evaluated composite resin.

## Discussion

Investigating the physical and mechanical properties of composite resins directly influences the ability to predict their behavior under clinical conditions in the oral cavity; therefore, these biomaterials must exhibit mechanical behavior similar to that of dental tissues. The values reported in this study for elastic modulus and hardness fall within the parameters documented in the literature for dental tissues, such as enamel (80.94 GPa and 418 VHN) and deep dentin (17.06 GPa and 76 VHN) [15-17]. These benchmarks are closely reflected in the outcomes obtained for the three evaluated composite resins.

In the present study, the highest Vickers hardness value was achieved by the Filtek Z250 resin (128.98 VHN), a microhybrid composite containing a 75-85 wt% filler loading and incorporating secondary particles such as zirconia. This outcome demonstrated a superior performance compared to that reported by Rastelli in 2012 (72.6 VHN) for the same material. This discrepancy is likely associated with a lower degree of conversion of the monomer matrix in the previous study, as those samples were photopolymerized using a curing unit with a lower irradiance (400 mW/cm^2^) than the one employed herein [18].

Regarding the performance of nanohybrid composites during hardness testing, the New Stetic resin achieved a value (110.93 VHN) similar to that reported by Randolph in 2016, where the highest mean value was observed for the nanohybrid composite Clearfil Majesty (Kuraray; 108.3 VHN). Discrepancies were noted regarding their filler loadings - 70.80 wt% for New Stetic versus 82 wt% for Clearfil - as well as particle shape and distribution. The superior behavior of the New Stetic resin may be attributed to its Nano Smart Position (NSP) technology, wherein the incorporation and distribution of spherical nanoparticles confer enhanced resistance to indentation [18-21].

A statistically significant difference was observed with respect to Empress Direct (64.61 VHN). Although this resin falls within normal parameters, its performance in this test may be associated with differences in chemical composition, particle distribution, and particle morphology. Its filler loading ranges from 77.5% to 79%, with a high proportion of glass particles within its composition, which confers superior aesthetic properties [20]. It is important to note that the incremental thicknesses recommended by manufacturers should be strictly followed to achieve the expected clinical outcomes. However, according to Lombardini et al., nanoparticle-reinforced composites exhibit less alteration in indentation resistance compared to microhybrid resins at layer thicknesses of 2-3 mm [22].

The depth of cure test demonstrated results within the quality parameters for all three materials. Filtek Z250 achieved complete polymerization at a layer thickness of up to 2.87 mm, followed by the New Stetic experimental resin with a depth of cure of 2.64 mm. This performance can be understood given the similarity in the monomer composition of their organic matrices, such as the inclusion of the Bis-EMA molecule, which exhibits favorable properties including low viscosity, low water sorption, and low polymerization shrinkage. Conversely, while the use of monomers such as triethylene glycol dimethacrylate (TEGDMA) can increase polymerization shrinkage, maintaining a balanced ratio with other monomers, such as urethane dimethacrylate (UDMA), enhances the degree of conversion of the polymeric matrix, thereby promoting superior mechanical performance. Empress Direct achieved complete polymerization at a layer thickness of 2 mm. Its polymerization behavior is driven by the inclusion of spherical and irregular subangular nanofiller particles, which yield low polymerization shrinkage and allow for better light refraction compared to the particles found in microhybrid resins. Finally, utilizing a light-curing unit with an appropriate wavelength that matches the specific photoinitiator remains a critical factor for achieving complete monomer conversion during photopolymerization [23-25].

The analysis of the elastic modulus indicates no statistically significant differences, yet distinct values for each resin: New Stetic (3,377.5 MPa), Filtek Z250 (2,401.3 MPa), and Empress Direct (2,277.3 MPa) [26]. Variations in mechanical properties are linked to filler loading and the specific distribution of inorganic filler particles and monomers, with nanoclusters in certain resins providing reduced inter-matrix tension [18,20,21,24].

This in vitro study was conducted following the parameters and guidelines specified in the technical standards for each test, thereby ensuring a controlled environment; however, given the positive results obtained with the test resin, the control of variables in these tests may be considered a study limitation. Further testing is required to evaluate dynamic factors specific to the oral cavity, such as hydrolytic degradation, fatigue under cyclic loading, or optical stability over time, in order to interpret the long-term behavior of this resin. We suggest conducting further in vitro research with larger sample sizes and broader multi-property analyses, as well as subsequent in vivo testing.

## Conclusions

Within the limitations of the study, it can be concluded that significant differences were observed among the three composite resins analyzed regarding hardness and depth of cure. Conversely, no statistically significant differences were observed in the elastic modulus, despite variations in the numerical values ​​recorded across the materials. The experimental New Stetic composite met all established technical standards, showing performance comparable to that of Filtek Z250 and Empress Direct. It demonstrated superior elastic modulus and hardness values ​​while remaining within standardized thresholds for depth of cure.
